# Undifferentiated Pleomorphic Sarcoma of the Proximal Thigh With Neoplastic Fever and Metachronous Sternal Metastases: A Case Report and Review of the Literature

**DOI:** 10.7759/cureus.72401

**Published:** 2024-10-25

**Authors:** Abner J Cruz-Alvarenga, Carlos F Bendaña Laínez, Karla V Villalta Salgado

**Affiliations:** 1 Department of Medicine, Universidad Nacional Autónoma de Honduras, Tegucigalpa, HND; 2 Department of Pathology, Instituto Hondureño de Seguridad Social, Tegucigalpa, HND

**Keywords:** fever of unknown origin (fuo), metachronous metastases, neoplastic fever, soft tissue sarcoma, undifferentiated pleomorphic sarcoma (ups)

## Abstract

Undifferentiated pleomorphic sarcoma (UPS) is a rare subtype of undifferentiated soft tissue sarcoma (USTS). UPS is a fast-growing malignant tumor with a high mitotic rate and a high risk of distant metastasis that increases proportionally to tumor size. Obtaining an early diagnosis confers a more favorable prognosis. Neoplastic fever (NF) is the refractory fever subsumed under the category of classic fever of unknown origin (FUO) caused by malignant neoplasms. NF is a paraneoplastic syndrome that rarely occurs in STS. Among UPS tumors, there is an exceptional subtype that causes refractory fever and signs of systemic inflammation. The coexistence of UPS with NF (UPS-NF) represents an unusual event. We present an exceptional case of a 58-year-old male patient with a two-month history of FUO that revealed an atypical clinical presentation of NF generated by a rare STS of the anterior thigh with subsequent sternal metastases. The patient received neoadjuvant chemotherapy and underwent a compartmentectomy with a microscopic residual tumor (R1), followed by postoperative adjuvant radiotherapy with unfavorable results. The patient also presented overlapping paraneoplastic manifestations (NF and areolar hyperpigmentation) that occur concurrently with the onset of metachronous sternal metastases, highlighting the uninvolved lung parenchyma.

## Introduction

Soft tissue sarcoma (STS) is a group of malignant tumors that encompasses all mesenchymal neoplasms arising from the connective tissues [1,2]. The annual incidence of STS is approximately 50 cases per million people [3], accounting for less than 1% of all malignant tumors [3,4]. Due to metastatic tropism to the lungs [1,3], roughly 10% of patients with STS have detectable metastases at diagnosis of the primary tumor [3]. Although no definitive etiologies have been identified [5], at least 25% of radiation-associated STSs are undifferentiated [3].

Undifferentiated pleomorphic sarcoma (UPS) is a high-grade aggressive STS [3,6]. UPS was included in the malignant tumors of uncertain differentiation of the 2020 WHO Classification of STS [6]. UPS is a subtype of undifferentiated STS (USTS) [3]. UPS was previously known as malignant fibrous histiocytoma, first described in 1964 [7], and reclassified in 2002 by the WHO Classification of STS as UPS [2,3,7], because, according to its tumorigenesis, UPS arises from a subpopulation of cells called side population cells [6], rather than histiocytes, as previously thought [1,2,6]. USTSs have a ubiquitous anatomical distribution [3,7].

Currently, USTSs are a heterogeneous group of rare malignant neoplasms whose diagnosis remains one of exclusion [3,8]. Therefore, the diagnosis of UPS is performed excluding other well-classified STSs [3,6]. UPS is a rare subtype characterized by a lack of specific immunohistochemical markers for a determined lineage differentiation [3,4]. Its cellular heterogeneity is the key distinguishing feature because it harbors tumor cells with a marked cellular pleomorphism composed of different cell types with undifferentiated morphology [1,9].

UPS with neoplastic fever (UPS-NF) is a specific subtype of UPS that presents some clinical manifestations that differentiate it from the other subtypes [9,10]. NF is defined as refractory fever caused by the tumor cells themselves [9,11]. NF is also known as fever of unknown origin (FUO) [9,10] because FUO induced by malignant neoplasms (12.8%) belongs to the category called “classic FUO” [5,12]. In 1961, Petersdorf and Beeson defined FUO as a temperature of ≥ 38.3 °C for at least three weeks, which evades diagnosis despite one week of inpatient investigations [5,12,13]. Durack and Street shortened the third criterion of a period to three days of inpatient investigations or three outpatient visits [12]. One week of hospitalization is no longer considered indispensable [13].

The mainstay of treatment for localized UPS is surgical excision with microscopically negative margins combined with radiotherapy (RT) [3,6,14]. RT has been associated with better control over local recurrence [1-3]. The use of chemotherapy is controversial [3,5]. UPS is a chemoresistant tumor [15]. Chemotherapy is preferred for unresectable tumors [6,9]. RT and chemotherapy are recommended as adjuvant therapies [1,3]. However, they do not influence overall survival [1,2]. UPS is sensitive to immunotherapy such as ipilimumab (anti-CTLA4), nivolumab (anti-PD1), and pembrolizumab (anti-PD1) and is the current treatment approach [1,6,10].

The combination of local resection and RT has survival and recurrence rates similar to those of amputation, with the added benefit of functional preservation of the affected limb [5]. Amputations are reserved as an extreme measure since overall survival rates were not higher than limb-sparing surgery [6]. The overall five-year survival rate for UPS is 60% for all ages [14]. The median overall survival for late-stage UPS (metastatic disease) is approximately one year (8-12 months) [9,14]. Since the initial diagnosis of UPS, roughly 30%-50% of all patients die within five years [14]. Therefore, experienced surgeons must obtain negative surgical margins to achieve a favorable prognosis [1,2,7]. The R0 resection margin is the most important favorable prognostic factor [1,2].

## Case presentation

**Figure 1:**
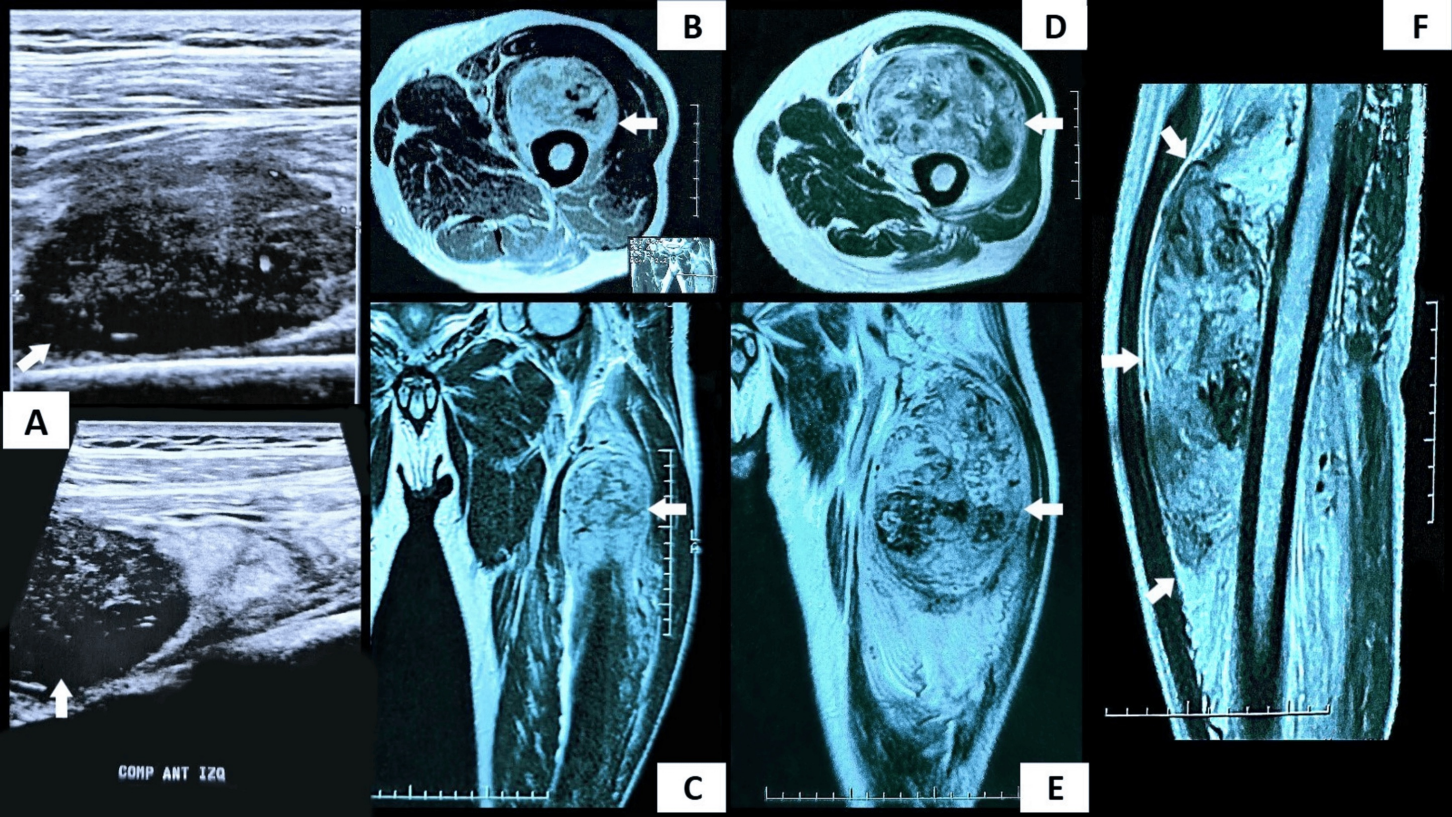
Sequence of images showing accelerated tumor growth during an 86-day period (2.8 months). Cross-sectional view: B and D. Coronal view: C and E. Sagittal view: F. (The tumor is indicated by white arrows). (A) The ultrasound initially shows a mass with dimensions of 2.9 × 4.5 × 5.1 cm (day 1).  (B-C) The MRI shows a mass with dimensions of 3.8 × 5.6 × 10 cm (day 13).  (D, E, and F) The MRI shows a tumor growth of 8.8 × 9.9 × 13.5 cm that persists after neoadjuvant chemotherapy (day 86).

This is a case of a 58-year-old male patient with personal pathological antecedents of diabetes and arterial hypertension. The patient presented a two-month medical history of FUO and a subsequent association with progressive pain in the left lower limb that manifested 45 days after the onset of fever. This case revealed an atypical presentation of a patient who presented fever as the clinical debut of a rare STS. The primary tumor originated in the vastus intermedius muscle in the anterior compartment of the left side. Refractory fever caused by an occult tumor revealed a diagnostic challenge of obscure etiology because the transient coexistence of COVID-19 initially masked the NF caused by the sarcoma. The fever was attributed to viral infection; however, after the coronavirus-induced clinical symptoms resolved, the fever persisted in an erratic pattern with fluctuating temperatures ranging from 38.2 to 39.4°C. Physical examination was of great relevance, which demonstrated a mass of considerable size in the left proximal thigh; the mass was perceived as apparently adhered to deep tissues based on palpation. The ultrasound showed findings of a mass of 2.9 × 4.5 × 5.1 cm with features of malignancy (Figure 1A). Therefore, the patient was referred to the oncology department two months after the onset of the fever. The MRI revealed a solid lesion of 3.8 × 5.6 × 10 cm, with a circumscribed inflammatory process in the perifacial tissues with areas of central necrosis confined to the neoplastic tissue (intratumoral). Bony and vascular structures were shown unscathed; no locoregional lymphadenopathies were found (Figure 1B-1C). Chest radiographs and CT were normal.

**Figure 2:**
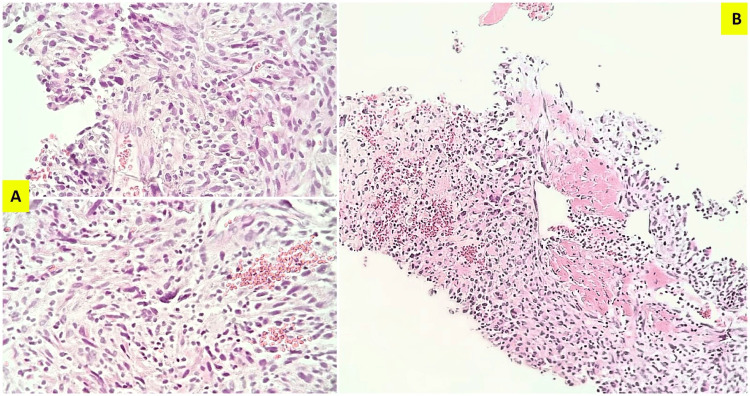
Undifferentiated pleomorphic sarcoma with hematoxylin and eosin (H&E) staining. (A) Sections of the fibrous connective tissue and striated muscle obtained from the tumor in the anterior thigh showing mesenchymal proliferation composed of numerous atypical spindle-shaped cells with some bizarre multinucleated tumor giant cells characterized by pleomorphic nuclear atypia and atypical mitotic figures arranged in a storiform and fascicular pattern (H&E, x20). (B) Atypical mesenchymal-looking spindle-shaped cells, infiltrating striated muscle fibers, and tumor necrosis with prominent heterogeneously distributed vascularization (H&E, x10).

The hematologic examinations and the set of investigations performed sequentially to rule in or rule out possible etiologies did not reveal any pattern of infection; however, his CRP was elevated (10.4 mg/dL; reference range (conventional units), 0-0.3 mg/dL), and the biomarkers of renal injury and function had abnormal laboratory values: serum creatinine, 1.70 mg/dL (reference range (conventional units) for male, 0.6-1.2 mg/dL; International System of Units (SI) reference, 53-106 µmol/L); 24-hour urine protein, 246.8 mg/24 hours (reference range (conventional units), <150 mg/24 h; SI reference, <0.15 g/24 h); blood urea nitrogen, 25.98 mg/dL (reference range (conventional units), 7-18 mg/dL; SI reference, 2.5-6.4 mmol/L); and serum uric acid, 7.50 mg/dL (reference range (conventional units) for male, 3.1-7.0 mg/dL; SI reference, 0.18-0.41 mmol/L). Core needle biopsy revealed cylinders of fibrous connective tissue and striated muscle infiltrated by atypical mesenchymal-looking spindle-shaped cells with marked pleomorphism, bizarre tumor cells, atypical mitoses, and tumor necrosis, accompanied by giant cells and heterogeneous lax areas with secondary elements, including xanthomatous cells. The tumor specimen contained certain highly vascularized areas. Histopathological features using H&E staining revealed USTS and were most distinctive with an undifferentiated high-grade pleomorphic sarcoma as a preliminary diagnosis when considering differential diagnoses (Figure 2). Subsequent to histopathological diagnosis, immunohistochemical investigations revealed that CD68 and vimentin were positive, while desmin, S100, and myogenin were all negative. Immunohistochemical features were consistent with USTS, subtype UPS, thus confirming the pathological diagnosis.

**Figure 3:**
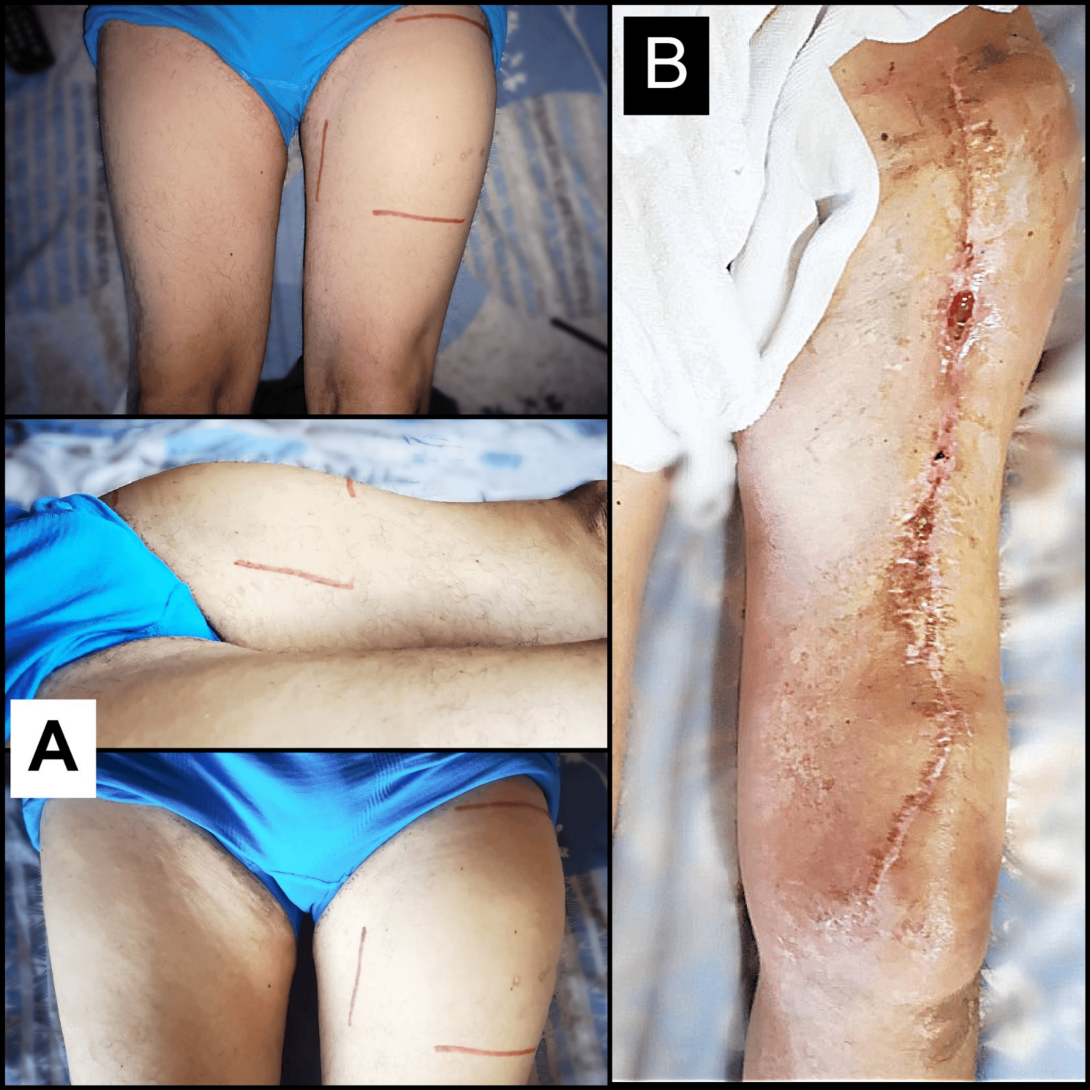
Limb-sparing compartmentectomy. (A) Preoperative images of the patient with a mass in the left proximal thigh. (B) Postoperative view of the compartmentectomy with resection of the anterior compartment that includes the entire sartorius muscle and the quadriceps muscle of the left thigh.

Retroperitoneal lymphadenopathy of uncertain significance was evidenced on CT in the absence of abdominal tumors (“coal-mine canaries”). In order to reduce the tumor size, neoadjuvant chemotherapy was decided, performing two cycles over a period of 44 days prior to surgery. Follow-up physical examination showed an increase in mass size, along with an intensification of compression symptoms: pain and weakness of the limb. A second oncologist evaluated the patient and decided to perform a second MRI, which revealed an aggressive tumor with a rapid growth pattern, extension of peritumoral edema, and areas of highly vascularized necrosis with tumor enlargement in all its dimensions (8.8 × 9.9 × 13.5 cm) (Figure 1D-1F). Despite treatment, it highlighted an increase in the primary tumor size of up to 8.4 cm. Consequently, it was decided to discontinue the chemotherapy regimen due to an inadequate response to treatment, and the patient was rescheduled to undergo urgent surgery. The patient suffered loss of limb function because reconstructive plastic surgery was not performed after oncological resection (Figure 3). After surgery, there was a gradual regression of renal injury biomarkers, the fever disappeared, and the CRP was also normalized.

**Figure 4:**
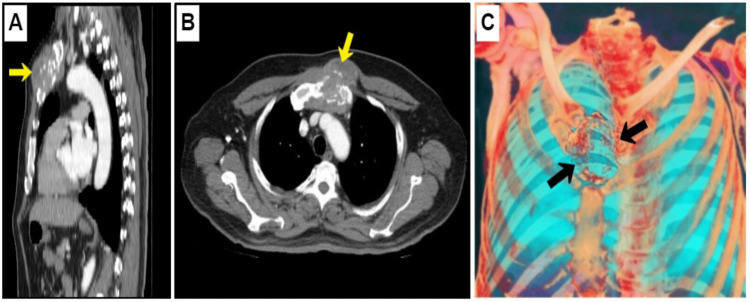
Computed tomography (CT) of the chest. (A) Sagittal view. (B) Cross-sectional view. Contrast-enhanced chest CT: (A-B) shows metachronous bone metastases in the sternum of 5.6 x 5.9 x 7.2 cm (yellow arrows). Three-dimensional CT image: (C) shows erosion and destruction of the sternal manubrium by expansive tumor growth (black arrows).

**Figure 5:**
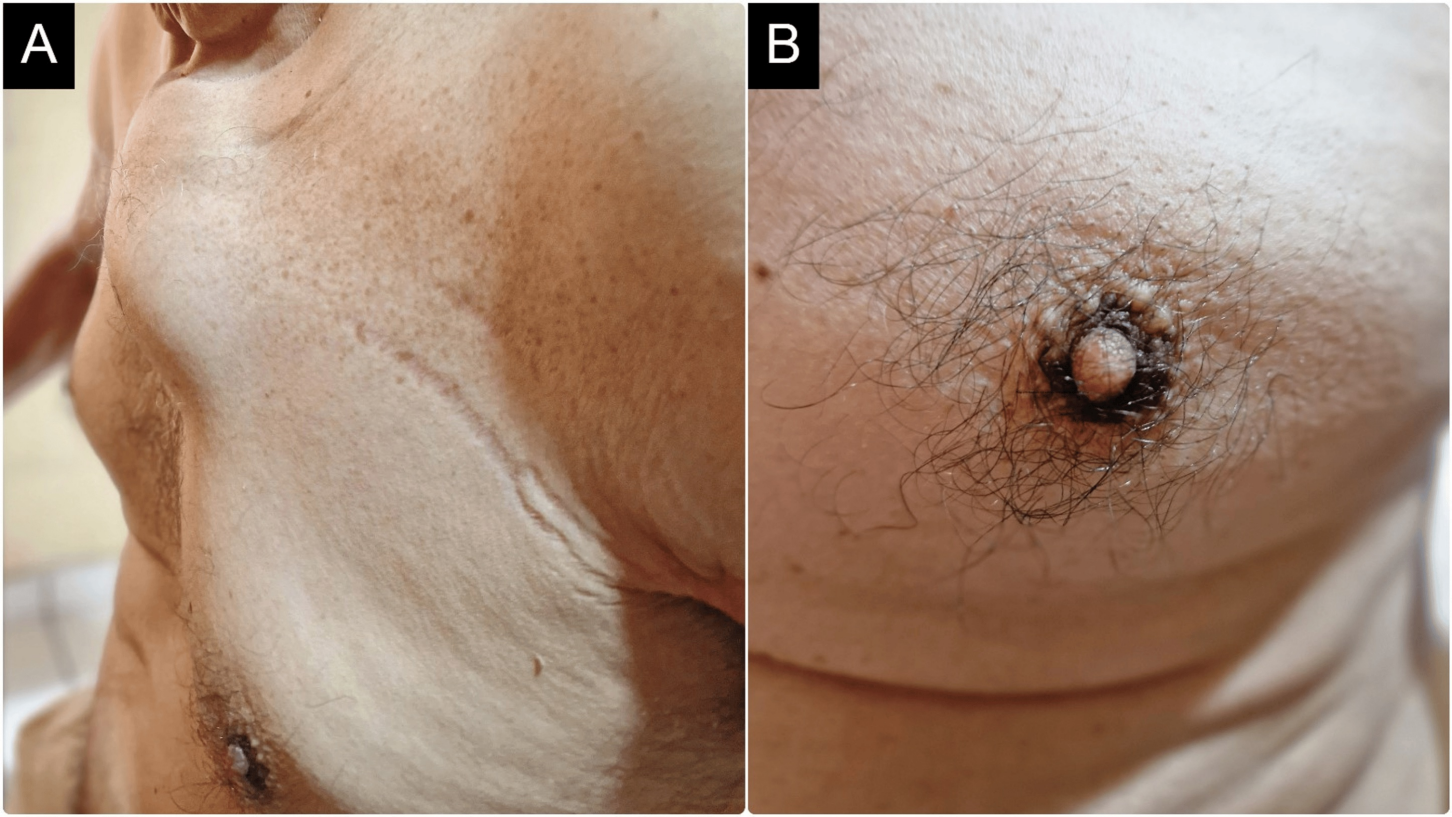
Metachronous bone metastases. (A) Metachronous sternal metastases arising from the undifferentiated pleomorphic sarcoma. (B) Paraneoplastic hyperpigmentation of the areola.

The anatomopathological report of the completely excised tumor performed by a second pathologist reaffirmed the diagnosis of high-grade UPS (grade 3) and also showed the absence of lymphovascular and perineural invasion. However, it revealed an R1 resection margin (microscopic residual tumor). Therefore, the patient received adjuvant radiotherapy, although it was administered with delay four months after surgery due to surgical site infection. Finally, a postradiation fracture of the proximal femur was presented as a late complication, three months after radiotherapy. Although CT did not report metastases to the thoracoabdominal organs and regular checkups were normal for more than a year (13 months), the patient went to the emergency room for precordial pain and fever and was eventually diagnosed with distant metastases. Chest CT revealed a solid tumor in the sternum due to metachronous metastases; additionally, CT reported the absence of lung parenchymal metastases (Figure 4). Of note, the patient presented again with NF in addition to bilateral hyperpigmentation of the areolas as heraldic signs of the onset of metastases (Figure 5). Finally, ubiquitous osteolytic bone metastases developed. No metastasectomy was performed; instead, the patient underwent palliative radiotherapy. The patient died after 7.5 months of metastases, two years after the initial diagnosis.

## Discussion

The annual incidence of UPS is extremely low, and the UPS-NF subtype is an exceptionally rare disease [9]. The diagnosis and treatment of UPS are highly challenging due to the pathological classification and the genomic complexity of USTSs [1,8]. UPS is a high-grade STS, which usually has a poor prognosis [1,2,16], because it is a tumor prone to local recurrences with an elevated risk of distant metastases compared to other types of STS [1,2].

Clinically, UPS usually presents as an asymptomatic, unremarkable, rapidly growing cutaneous or subcutaneous nodule without overlying epidermal changes [6]. The classical presentation of UPS includes an aggressive, deep-seated tumor with progressive and painless growth [1]. Physicians often exclude STS from differential diagnoses because they are rare. Patients often do not seek prompt care due to the typically painless presentation of these tumors; therefore, delays in the diagnosis and treatment of UPS are extremely common [4]. In our patient, the mass was painful and accompanied by NF.

NF is a rare paraneoplastic syndrome [11]. Malignant neoplasms may cause NF usually associated with aseptic elevation of inflammatory parameters (patient’s CRP level was 10.4 mg/dL), a presentation that occurs in only 2.3% of patients [9,10], and only a small fraction of this percentage is due to STS [9]. NF may be caused by the malignant process itself or triggered by an associated paraneoplastic syndrome [13]. There may be doubts about the association between fever and UPS; consequently, treatment may be delayed due to the lack of understanding that these symptoms may be a clue to diagnosis [16].

NF is an unusual clinical manifestation in patients with bone and STSs, and only a few cases have been reported to date [11]. In the scientific literature, there are exceptional cases of UPS that initially presented with fever [7,9,10,15]. Currently, only one retrospective study has focused specifically on this rare subtype of UPS-NF and revealed that the incidence of NF in patients with UPS was only 3.83% [9]. In a cohort of 195 patients with bone and STSs, only 5.5% presented NF as a manifestation of a paraneoplastic syndrome, and only in three cases was NF caused by primary tumors [5,11]. In this case report, the NF was initially caused by the primary tumor in the absence of metastases.

Patients with the UPS-NF subtype may have a better prognosis, have shown lower metastatic rates (14.29% vs 44.94%), and have a higher three-year survival rate (85.71% vs 59.55%) than UPS without NF [9]. In this report, the patient developed distant metastases 13 months after surgery with a survival of 24 months. NF reappeared again along with metachronous metastases as an early manifestation of relapse. The most common site where UPS metastasizes is in the lungs, while the bones are a less frequent site [1,3,4]. The concurrence of sternal metastases with the absence of metastases to the pulmonary parenchyma is extremely rare. High-grade, deep-seated, stage T3 tumors due to delay in surgery and R1 resection were factors associated with a poor prognosis in this case [1-4,14].

If the objective is to reduce the primary tumor size with preoperative chemotherapy, and given that immunotherapy was not available, cross-checking of references with the outcome of this case report will be futile, the tumor will not stop growing, and the surgery will be delayed. Neoadjuvant chemotherapy should be avoided in nonsensitive histotypes [1-3,5,10,15]. Tumor size ≥5 cm and R1/R2 resection margin are prognostic markers of poor overall survival [1]. Surgery should be performed when the tumor size remains small (<5 cm). In aggressive high-grade sarcomas, early diagnosis and treatment are crucial, as the risk of metastasis increases almost linearly as the tumor enlarges [1-4,14].

Pathophysiology

The tumor microenvironment plays an essential role in the development of USTS [8]. A causal association according to histopathology has been suggested between the tumor microenvironment and systemic inflammation [7]. The production and release of pyrogenic cytokines (in situ production of febriferous substances by the tumor) or spontaneous tumor necrosis are the likely origins of NF [9,12]. Wang et al. suggest that NF may be caused by intratumoral necrosis and peritumoral inflammation [9]. Tumor growth may cause underlying tissue inflammation [9,11]. The UPS-NF subtype is typified by distinctive findings on the MRI: intratumoral necrosis and extensive peritumoral edema of the soft tissues [9].

Tumor cells may increase abnormal production of inflammatory proteins [11]. Hashimoto et al. suggested that inflammatory cytokines may play a role in the tumor microenvironment [7]. Cytokines, such as granulocyte colony-stimulating factor (G-CSF), have been proposed as markers for UPS [16]. Active cytokine production from primary tumors has previously been described as an atypical presentation of UPS accompanied by fever and signs of systemic inflammation [10,15,16]. G-CSF is thought to cause a paraneoplastic syndrome that occurs with fever or increased CRP [16]. Neoplastic production of IL-6, IL-7, IL-8, SCF, TGB, and GM-CSF has also been suggested [7,15].

The naproxen test can be used to suggest a malignant etiology of fever, as NF defervescence is often resistant to other antipyretics [11-13]. Total excision of the tumor in STSs can achieve permanent lysis of the fever [11]. Inflammation markers and body temperature have been reported to decrease dramatically after surgery in UPS tumors [7,9,16]. As discussed in this report. In some cases, patients with UPS with intratumoral necrosis and peritumoral edema do not present NF; this suggests that NF is conditioned by two factors: thermogenic factors generated by the tumor itself and the interindividual variability of the host’s response to these thermogenic factors [9].

Fever is a complex physiological response orchestrated primarily by the preoptic area and anterior hypothalamus and modulated by multiple thermoeffectors in the peripheral tissues. Endogenous pyrogens and endogenous cryogens exert their biological functions to achieve thermal homeostasis during temperature set points: fever, defervescence, anapyrexia, and euthermia. The febrile response is mediated mainly by prostaglandin E2, which stimulates the thermoregulatory center in the preoptic area of the hypothalamus to produce an upward shift of the core temperature, thus triggering the febrile response [12]. However, the specific mechanism of NF has not yet been elucidated and requires further research [9].

## Conclusions

The extremely low incidence and incipient natural history of UPS make these tumors often underdiagnosed. The refractory pattern of NF can play a prognostic role by acting as a clinical clue that leads to an early diagnosis of rare types of sarcomas with undifferentiated cell lines. Refractoriness may be a hallmark of malignancy. In this clinical case, we describe the association of concomitant NF with other paraneoplastic manifestations. Metachronous bone metastases to the sternum without metastasizing to the lung parenchyma is an extremely rare presentation of UPS.
